# 37 Subtype frequencies and demographic characteristics of juvenile idiopathic arthritis in Batna, Algeria

**DOI:** 10.1093/rheumatology/keac496.033

**Published:** 2022-10-07

**Authors:** Djohra Hadef, Samy Slimani, Mohamed Choukri Khamari, Walid Mekaoussi, Alexandre Belot, Pierre Quartier

**Affiliations:** 1 Department of Pediatrics, University Hospital Center of Batna, Faculty of Medicine, Batna 2 University; 2 Atlas Clinic of Rheumatology; 3 Private Clinic of Rheumatology, Batna, Algeria; 4 Pediatric Nephrology, Rheumatology, Dermatology Unit, Hôpital Femme Mère Enfant, Hospices Civils de Lyon, National Referee Centre for Rheumatic and AutoImmune and Systemic diseases in children (RAISE), Lyon, France; 5 Pediatric Immunology-Hematology and Rheumatology Unit Necker Hospital, Assistance Publique Hôpitaux de Paris, National Referee Centre for Rheumatic and AutoImmune and Systemic Diseases in Children (RAISE), Paris, France

## Abstract

**Background:**

Juvenile idiopathic arthritis (JIA) is the most common chronic rheumatic disease in childhood. There is a disparity in the prevalence of Juvenile idiopathic arthritis (JIA) subsets between different geographical areas or ethnic groups. In Arabic and African populations, data describing JIA are scarce. However, the epidemiological studies remain the best tool to understand the disease and to improve its management.

**Objectives:**

To determine subtype, frequency, demographic and clinical features of JIA in Batna -Algeria- and to compare the findings with other JIA populations worldwide.

**Methods:**

A multicentre retrospective descriptive study was conducted in Batna health centers (public and private sectors), over a seven-year period from January 2013 to December 2019, based on data collected on JIA patients. As public sector source, we referred to the department of pediatrics of the university hospital center (CHU Benflis Touhami Batna), and as private sector source, we referred to private adult rheumatologists based in Batna. The studied variables were: gender, age at the initial symptoms, age at diagnosis, JIA subtype based on International League of Associations for Rheumatology (ILAR) criteria, symptoms at onset, disease duration at the latest follow up, presence of uveitis, auto antibodies (antinuclear antibodies, Rheumatoid Factor and anti-CCP) pattern, joint imaging results, JIA status at the time of enrolment and the latest follow-up.

**Results:**

The study included a total of 69 cases of JIA that were being followed in Batna health centers over the study period. The female to male ratio was 1.83. The median age at diagnosis was 9 years (range 1–16). Forty-six patients (72%) were diagnosed within the first year after disease onset. At the latest follow-up, the median disease duration onset was 1 year (range 1–8 years). There were 34 oligoarthritis (49.3%), 9(13%) rheumatoid factor (RF) negative polyarticular JIA, 8(11.6%) RF positive polyarticular JIA, 6(8.7%), systemic arthritis, 6(8.7%) enthesitis-related arthritis, 3(4.3%) psoriatic arthritis and 3(4.3%) undifferentiated arthritis. Nine patients (18.7%) were anti-nuclear antibody (ANA) positive, and 21 patients (30.4%) had indeterminate ANA status.

**Conclusion:**

Oligoarthritis was the most common JIA subtype in our study. The RF positive polyarthritis frequency was higher than in literature. Prospective multicentre studies are necessary to better identify the JIA peculiarities in our country.

**Disclosure of Interest:**

None declared

